# A Nationwide Survey and Multicenter Registry-Based Database of Cerebral Autosomal Dominant Arteriopathy With Subcortical Infarcts and Leukoencephalopathy in Japan

**DOI:** 10.3389/fnagi.2020.00216

**Published:** 2020-07-14

**Authors:** Akihiro Shindo, Ken-ichi Tabei, Akira Taniguchi, Hiroaki Nozaki, Osamu Onodera, Akihiko Ueda, Yukio Ando, Takao Urabe, Kazumi Kimura, Kazuo Kitagawa, Haruo Hanyu, Teruyuki Hirano, Hideaki Wakita, Hidenao Fukuyama, Tatsuo Kagimura, Yoshihiro Miyamoto, Misa Takegami, Satoshi Saito, Akiko Watanabe-Hosomi, Ikuko Mizuta, Masafumi Ihara, Toshiki Mizuno, Hidekazu Tomimoto

**Affiliations:** ^1^Department of Neurology, Mie University Graduate School of Medicine, Tsu, Japan; ^2^Department of Dementia Prevention and Therapeutics, Mie University Graduate School of Medicine, Tsu, Japan; ^3^Department of Neurology, Clinical Neuroscience Branch, Brain Research Institute, Niigata University, Niigata, Japan; ^4^Department of Neurology, Graduate School of Medical Sciences, Kumamoto University, Kumamoto, Japan; ^5^Department of Amyloidosis Research, Nagasaki International University, Nagasaki, Japan; ^6^Department of Neurology, Juntendo University Urayasu Hospital, Chiba, Japan; ^7^Department of Neurology, Graduate School of Medicine, Nippon Medical School, Tokyo, Japan; ^8^Department of Neurology, Tokyo Women’s Medical University, Tokyo, Japan; ^9^Department of Geriatric Medicine, Tokyo Medical University, Tokyo, Japan; ^10^Department of Stroke and Cerebrovascular Medicine, Kyorin University, Tokyo, Japan; ^11^Department of Internal Medicine, Nanakuri Memorial Hospital, Fujita Health University, Tsu, Japan; ^12^Center for the Promotion of Interdisciplinary Education and Research, Kyoto University, Kyoto, Japan; ^13^Translational Research Center for Medical Innovation, Foundation for Biomedical Research and Innovation at Kobe, Kobe, Japan; ^14^Center for Cerebral and Cardiovascular Disease Information, National Cerebral and Cardiovascular Center, Osaka, Japan; ^15^Department of Preventive Medicine and Epidemiologic Informatics, National Cerebral and Cardiovascular Center, Osaka, Japan; ^16^Department of Neurology, National Cerebral and Cardiovascular Center, Suita, Japan; ^17^Department of Neurology, Graduate School of Medical Science, Kyoto Prefectural University of Medicine, Kyoto, Japan

**Keywords:** small vessel disease, CADASIL, NOTCH3, risk factors, migraine, microbleeds

## Abstract

**Objectives:**

Clinical characteristics of cerebral autosomal dominant arteriopathy with subcortical infarcts and leukoencephalopathy (CADASIL) include migraine, recurrent stroke, white matter lesions, and vascular dementia. CADASIL is one of the most common hereditary cerebral small vessel diseases. Clinical presentation of CADASIL varies and a racial gap may exist between the Asian and Caucasian populations. This is the first nationwide epidemiological survey which aimed to elucidate the clinical features of CADASIL in Japan. Moreover, the registration database of CADASIL was constructed.

**Methods:**

Subjects included CADASIL patients who visited the hospitals (totally 1,448 hospitals) certified by the Japanese Society of Neurology and/or Japan Stroke Society in 2016. This study consisted of a two-step survey; patients with CADASIL were identified genetically by the first questionnaire, and their clinical features were assessed by the second questionnaire. Selected 6 hospitals registered the data of all CADASIL patients using a Research Electronic Data Capture (REDCap) system for the second questionnaire.

**Results:**

Based on the criteria, 88 patients (50 male and 38 female) with CADASIL were enrolled. The mean age of symptom onset was 49.5 years. Sixteen (18.2%) patients had an elderly onset (>60 years). Thirteen patients (13.6%) had history of migraine with aura and 33 patients (37.5%) had vascular risk factor(s). From among the 86 patients who were examined using magnetic resonance imaging, abnormal deep white matter lesions were detected in 85 patients (98.8%), WMLs extending to anterior temporal pole in 73 patients (84.9%), and cerebral microbleeds in 41 patients (47.7%). Anti-platelet therapy was received by 65 patients (73.9%). Thirty-eight patients (43.2%) underwent treatment with lomerizine hydrochloride. Thirty-four different mutations of *NOTCH3* were found in exons 2, 3, 4, 5, 6, 8, 11, 14, and 19. Most of the mutations existed in exon 4 (*n* = 44, 60.3%). The prevalence rate of CADASIL was 1.20 to 3.58 per 100,000 adults in Japan.

**Conclusion:**

This questionnaire-based study revealed clinical features and treatment status in Japanese CADASIL patient, although it may not be an exhaustive search. We have constructed the REDCap database for these CADASIL patients.

## Introduction

Cerebral autosomal dominant arteriopathy with subcortical infarcts and leukoencephalopathy (CADASIL) is an autosomal dominant disease and a common type of ischemic cerebral small artery disease and subcortical vascular dementia (Roman et al., 2002). First identified in 1996, mutations in the *NOTCH3* gene cause CADASIL and lead to small vessel arteriopathy in the central nervous system (Joutel et al., 1996). Patients with CADASIL commonly exhibit migraines with aura around at the age of 30 years, subcortical ischemic events at 50 years, and cognitive impairment between 50 and 60 years (Chabriat et al., 2009). Brain magnetic resonance imaging (MRI) displays extensive white matter hyperintensities including those in the anterior temporal lobe and external capsule. Although CADASIL is genetic small vessel disease, several reports have revealed the presence of vascular risk factors, such as hypertension, hyperlipidemia, and diabetes mellitus (Chabriat et al., 2009).

Although, Mizuta et al. (2017) found no significant difference in the clinical features of CADASIL between Japan and other countries, a nationwide survey in Japan was not conducted until 2019. The prevalence of migraines in Japanese CADASIL patients has been reported to be approximately 26.9–44.3% (Santa et al., 2003; Ueda et al., 2015; Mizuta et al., 2017; Koizumi et al., 2019), whereas a much lower prevalence of migraine has been reported in other Asian countries (Kim et al., 2006; Wang et al., 2011; Kang and Kim, 2015). While the clinical features of Asian CADASIL patients could be different from Caucasian patients (Kim et al., 2006; Wang et al., 2011; Choi et al., 2013; Kang and Kim, 2015), Japanese CADASIL patients might have other clinical characteristics.

Herein, this study aimed to elucidate the epidemiological and clinical features of Japanese CADASIL patients based on a Japanese nationwide survey. Moreover, we constructed a database for patients with CADASIL for future clinical studies.

## Materials and Methods

### Study Design and Subjects (Figure 1)

We conducted a two-step postal survey in 1,448 hospitals in all 47 Japanese prefectures. This study was carried out in accordance with the Declaration of Helsinki and approved by the Ethics Committee of the Mie University Graduate School of Medicine (permit number 2918).

**FIGURE 1:**
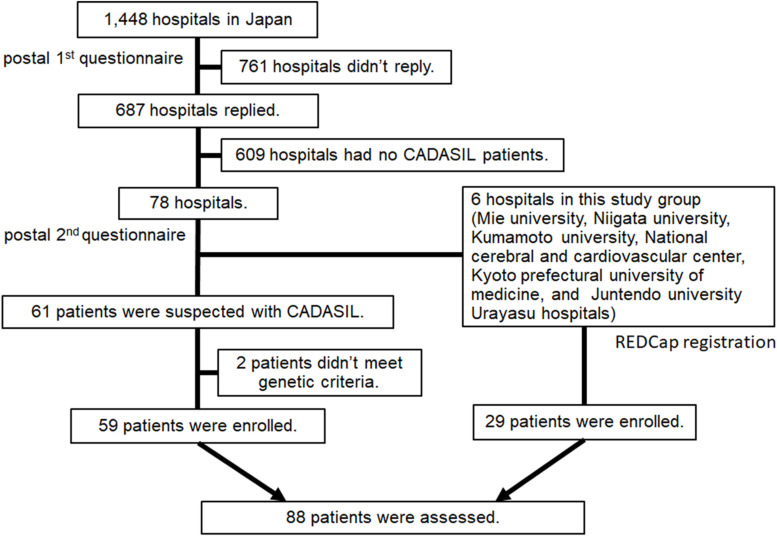
Flow diagram of study enrollment.

### The Two-Step Postal Survey

#### First-Step Postal Survey

Target subjects were CADASIL patients who visited the hospitals between April 1st, 2011 and June 30th, 2016. The hospitals surveyed were certified by the Japanese Society of Neurology and/or Japan Stroke Society in 2016 (in total 1,448 hospitals) and covered all 47 prefectures in Japan. In the first survey, a simple questionnaire asked whether there were any patients with CADASIL, and if so, how many.

#### Second-Step Survey

We obtained a response to the first questionnaires from 687 hospitals (47.4%). Of those, 78 hospitals had one or more patients with CADASIL and agreed to the next step. We sent a second questionnaire to the 78 hospitals to obtain further details. Detailed clinical information including age, sex, age at the onset (excluding migraine), height, weight, blood pressure (BP), birthplace, clinical presentations (stroke-like episodes, dementia, pyramidal sign, pseudobulbar palsy), migraine (with or without aura), ischemic stroke, hemorrhagic stroke, past medical history (hypertension, hyperlipidemia, diabetes mellitus, renal dysfunction, ischemic heart disease, and atrial fibrillation), type of the mutation of *NOTCH3* gene, family history, treatment, modified Rankin Scale (Japanese version) (Shinohara et al., 2006), findings of magnetic resonance imaging (MRI) including Fazekas scale (Fazekas et al., 1987), findings of single photon emission computed tomography (SPECT), and skin biopsy were requested. We received a response to the second questionnaire from 38 hospitals (48.7%) and 61 patients with cases defined as CADASIL were enrolled.

### REDCap Survey

We sent out a simple second-step questionnaire to six core hospitals, including Mie University Hospital, Niigata University Hospital, Kumamoto University Hospital, National Cerebral and Cardiovascular Center, Kyoto Prefectural University of Medicine Hospital, and Juntendo University Urayasu Hospital with questions regarding age, sex, age at onset, birthplace, and type of *NOTCH3* mutation. After the two-step postal survey, data for core hospitals were also collected using a Research Electronic Data Capture (REDCap) system (Harris et al., 2009). The REDCap database was built to elucidate the natural history, clinical features, and clinical studies for patients with CADASIL in Japan. After obtaining the consent and approval of the Local Institutional Review Board (IRB) Committee to participate, the physician sent a registration form to the secretariat at National Cerebral and Cardiovascular Center of Japan. Detailed data same as the second postal survey was obtained from REDCap. A written consent form was obtained from each patient. REDCap survey enrolled a total 29 patients with CADASIL and all data remained anonymous without any patients’ identifying clinical information.

### Diagnosis of CADASIL

The diagnosis of CADASIL was based on the genetic criteria that either the patient or a third-degree relative had *NOTCH3* mutations. Seventy-nine patients were identified to have *NOTCH3* mutations, and the other nine patients had the gene mutations present in their relatives.

### Statistical Analysis

Three types of prevalence rates of CADASIL: the most conservative estimate, the moderate estimate, and the most aggressive estimate, were estimated from the questionnaire sampling process and the Japanese population in 2017, i.e., 106,367,000 adults (over 18 years old) data from the Ministry of Internal Affairs and Communications.

The most conservative estimate was based on the assumption that there were no patients in the not answered site in the first survey, and that the number of patients of the not answered site in the second survey was at least one. The moderate estimate was based on the assumption that there were no patients in the not answered site in the first survey, and that the percentage of patients in the not answered site in the secondary survey was same that in the answered site. The most aggressive estimate was based on the assumption that the percentage of patients in the not answered site in the first survey was same that in the answered site, and that the percentage of patients in the not answered site in the secondary survey was same that in the answered site.

## Results

### Study Patients

A total of 90 patients with CADASIL were registered from 44 hospitals (38 hospitals by questionnaires and 6 hospitals using the REDCap system). Of those, we excluded 2 patients as they had no family history nor they filled the genetic diagnostic criteria. Data from 88 CADASIL patients were collected and analyzed (Figure 1).

### Age at the Onset and Geographic Data

**FIGURE 2:**
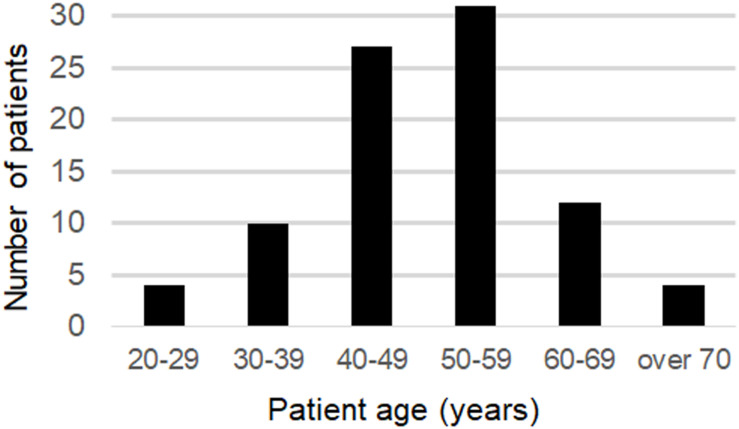
Distribution of ages at the onset.

The mean age at onset was 49.5 years old (20–76) for all patients and for the 58 patients (65.9%), the age of onset was between 40 and 59 years old. Sixteen of the patients had an onset over 60 years of age (18.2%), and 4 patients had an onset over 70 years of age (Figure 2).

**FIGURE 3:**
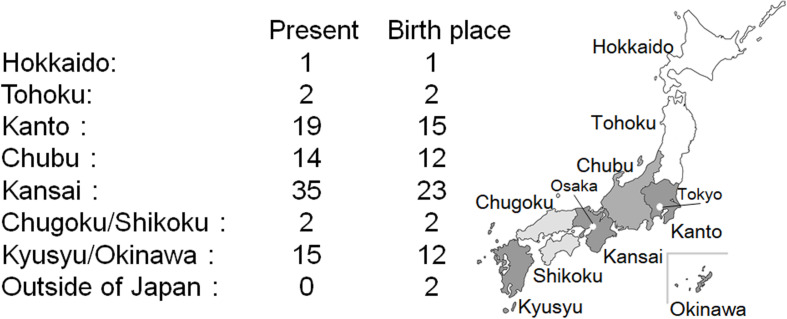
Current residence region and birthplace of CADASIL patients in Japan.

The current residence region and place of birth are shown in Figure 3. Kansai region has the highest number of CADASIL patients, followed by the Kanto and Kyusyu/Okinawa region. Birthplace tends to be the same as the current residence. Only a few patients with CADASIL were found in Hokkaido, Tohoku, and Chugoku/Shikoku regions. Two patients were born in out of Japan, and their birthplace was Taiwan.

### Demographic and Clinical Data in Japanese CADASIL Patients (Table 1)

Body mass index (BMI) was calculated using the patients’ height and weight, and the mean BMI was 22.8 kg/m^2^. Mean systolic BP was 120.0 mmHg, and mean diastolic BP was 74.2 mmHg. *NOTCH3* mutations were detected in 79 patients (89.7%) and 28 patients (31.8%) had a close family member with *NOTCH3* mutations. Nineteen patients were identified to have *NOTCH3* mutations in both the patients and their relatives. Skin biopsy was performed in 15 patients (17.0%), and granular osmiophilic material (GOM) was detected in 8 patients (9.1%). The detection rate of GOM was 53.3%.

**TABLE 1:** Demographic data in 88 Japanese CADASIL patients.

Demographics in 88 patients with CADASIL		
Mean age at onset (years)	49.5 ± 11.2	(20–76)
Male sex, (*n*, %)	50	62.5%
Body mass index (kg/m^2^)	22.8 ± 2.8	
**Blood pressure**		
Systolic (mmHg)	120.0 ± 13.6	
Diastolic (mmHg)	74.2 ± 9.8	
***NOTCH3* mutations**		
Patient (*n*, %)	79	89.7%
Close family (*n*, %)	28	31.8%
**Skin biopsy**		
Performed (*n*, %)	15	17.0%
Detected granular osmiophilic material (*n*, %)	8	9.1%
**Past history**		
Migraine (*n*, %)	25	28.4%
Migraine with aura (*n*, %)	12	13.6%
Hypertension (*n*, %)	13	14.8%
Dyslipidemia (*n*, %)	31	35.2%
Diabetes mellitus (*n*, %)	1	1.1%
One or more of above 3 vascular risk factors (*n*, %)	33	37.5%
Ischemic heart disease (*n*, %)	0	0%
**Clinical presentation**		
Stroke-like episode/Stroke (*n*, %)	59	67.0%
Dementia (*n*, %)	31	35.2%
Pyramidal sign (*n*, %)	41	46.6%
Pseudobulbar palsy (*n*, %)	20	22.7%
None of above (*n*, %)	2	2.3%
Modified Rankin Scale		
Excellent (0–2) (*n*, %)	59	67.0%
Moderate (3, 4) (*n*, %)	21	23.9%
Poor (5) (*n*, %)	5	5.7%
Death (6) (*n*, %)	2	2.3%
**Image findings**		
Magnetic resonance imaging (*n*, %)	86	97.7%
MRI findings (out of 86 patients)		
White matter lesion (*n*, %)	85	98.8%
Anterior temporal pole lesion (*n*, %)	73	84.9%
Fazekas grade 2 (*n*, %)	23	26.7%
Fazekas grade 3 (*n*, %)	56	65.1%
Microbleeds (*n*, %s)	41	47.7%
Single photon emission computed tomography (*n*, %)	40	45.5%
Cerebral blood flow reduction (% out of 40 patients)		
Frontal lobe (*n*, %)	25	28.4%
Temporal lobe (*n*, %)	22	25.0%
Parietal lobe (*n*, %)	26	29.5%
Occipital lobe (*n*, %)	7	8.0%
**Treatment**		
Anti-platelet therapy (*n*, %)	65	73.9%
Aspirin (*n*, %)	16	18.2%
Cilostazol (*n*, %)	42	47.7%
Clopidogrel (*n*, %)	16	18.2%
Dual anti-platelet therapy (*n*, %)	6	6.8%
Lomerizine hydrochloride (*n*, %)	38	43.2%

From past history, migraine was noted in 25 patients (28.4%) and migraine with aura in 12 patients (13.6%). Vascular risk factors including hypertension, dyslipidemia, and diabetes mellitus were detected in 13 (14.8%), 31 (35.2%), and 1 (1.1%) patients, respectively. There were 33 patients (37.5%) who possessed one or more of these vascular risk factors. None of the patients had any ischemic heart disease.

Stroke and the stroke-like episode was the most common symptom in 59 patients (67.0%) and dementia was reported in 31 patients (35.2%). Pyramidal sign and pseudobulbar palsy were detected in 41 (46.6%) and 20 (22.7%) patients, respectively. Two patients (2.3%) did not show these before mentioned symptoms. Modified Rankin scale outcome at the time of this investigation was excellent [0–2] in 59 (67.0%), moderate [3, 4] in 21 (23.9%), poor [5] in 5 (5.7%), and death [6] in 2 patients (2.3%).

Regarding the neuroimaging findings, brain MRI was performed in 86 patients (97.7%), from among whom, white matter lesions were detected in 85 patients (98.8%) and anterior temporal lobe lesions were detected in 73 patients (84.9%). Fazekas score 2 was reported in 23 patients (26.7%) and score 3 for 56 patients (65.1%). Cerebral microbleeds were detected in 41 patients (47.7%). Although, SPECT was performed in 53 patients (60.2%), findings suggestive of CADASIL were reported in only 40 patients (45.4%). Decreased in cerebral blood flow was detected 25 patients in frontal lobe (62.5%, out of 40 patients), in 22 patients in temporal lobe (55.0%), in 26 patients in parietal lobe (65.0%), and in seven patients in occipital lobe (17.5%).

Sixty-five patients (73.9%) underwent an antiplatelet therapy, 16 (18.2%) had aspirin, 42 (47.7%) had cilostazol, and 16 patients had (18.2%) clopidogrel. Dual antiplatelet therapy was given to 6 patients (6.8%). Thirty-eight patients were treated with lomerizine hydrochloride (43.2%).

### *NOTCH3* Mutations

Of the total 88 CADASIL patients, we confirmed *NOTCH3* gene mutations in 73 patients (83.0%) (Table 2). There was no description on the type of gene mutation in 15 patients. Thirty-four different mutations of *NOTCH3* were found in exons 2, 3, 4, 5, 6, 8, 11, 14, and 19. Most of the mutations existed in exon 4 (*n* = 44, 60.3%), and the most common mutations were p.R141C, p.R182C, p.R133C, and p.R153C. A novel mutation (p.C318Y) was found in one case.

**TABLE 2:** Summary of *NOTCH3* mutations in Japanese CADASIL patients.

Nucleotide change	Amino acid change	Exon	EGF-like repeat	Number of cases
c.163T>G	p.C55G	2	1	1
c.194G>C	p.C65S	2	1	2
c.224G>C	p.R75P	3	1	1
c.277T>G	p.C93G	3	2	1
c.316T>C	p.C106R	3	2	2
c.328C>T	p.R110C	3	2	1
c.397C>T	p.R133C	4	3	7
c.421C>T	p.R141C	4	3	10
c.431G>T	p.R144F	4	3	1
c.457C>T	p.R153C	4	3	5
c.505C>T	p.R169C	4	4	3
c.521_522delinsTG	p.C174L	4	4	2
c.544C>T	p.R182C	4	4	8
c.554G>A	p.C185Y	4	4	3
c.634T>C	p.C212R	4	5	1
c.635G>A	p.C212Y	4	5	1
c.665G>C	p.C222S	4	5	1
c.665G>A	p.C222Y	4	5	1
c.598_610delinsAGAACCC	p.Pro200_Ser204delinsArgThrPro	4	5	1
c.734G>A	p.C245Y	5	6	1
*c.953G>A	p.C318Y	6	8	1
c.969C>G	p.C323W	6	8	2
c.994C>T	p.R332C	6	8	2
c.1255T>C	p.C419R	8	10	2
c.1304G>A	p.C435Y	8	11	1
c.1370G>C	p.C457S	8	11	1
c.1630C>T	p.R544C	11	13–14	1
c.1703G>A	p.C568Y	11	14	1
c.1819C>T	p.R607C	11	15	1
c.2185T>G	p.C729G	14	18	1
c.3010T>G	p.C1004G	19	26	3
c.3045C>G	p.C1015W	19	26	1
c.3062A>G	p.Y1021C	19	26	2
c.3064T>G	p.C1022G	19	26	1
			Total	73

* c.953G>A (p.C318Y) identified as a novel mutation.

### Prevalence Rate of CADASIL in Japan

In terms of the most conservative estimate, 128 CADASIL patients were estimated and the prevalence rate was 1.20 per 100,000 adults. In terms of the moderate estimate, 180.6 CADASIL patients were estimated and the prevalence rate was 1.70 per 100,000 adults. In terms of the most aggressive estimate, 380.7 CADASIL patients were estimated and the prevalence rate was 3.58 per 100,000 adults. The prevalence rate of CADASIL in Japan was estimated to between 1.20 to 3.58 per 100,000 adults.

## Discussion

This is the first nationwide study for Japanese CADASIL patients. We show that the prevalence rate of migraines in Japanese CADASIL patients to be lower than European populations. The presence of vascular risk factors with hypertension and/or diabetes mellitus had a lower tendency than other European and Asian populations. The prevalence rate of CADASIL was 1.20 to 3.58 per 100,000 adults, and Kansai, Kanto, and Kyusyu/Okinawa regions had the largest distribution of CADASIL patients in Japan.

We also described a tendency of a low prevalence rate of migraine with aura in Japanese patients (13.6%) compared to the European counterparts. Table 3 shows the difference in CADASIL patients between Japan and other countries (Peters et al., 2004; Singhal et al., 2004; Kim et al., 2006; Herve et al., 2009; Adib-Samii et al., 2010; Wang et al., 2011; Pescini et al., 2012; Ciolli et al., 2014; Bianchi et al., 2015; Kang and Kim, 2015; Ueda et al., 2015; Nannucci et al., 2018; Koizumi et al., 2019). This table reveals a trend: a higher prevalence rate of migraine in European countries (34–75.3%) (Peters et al., 2004; Adib-Samii et al., 2010; Pescini et al., 2012; Ciolli et al., 2014; Bianchi et al., 2015; Nannucci et al., 2018), but a much lower prevalence in other Asian countries such as China and South Korea (4–8.7%) (Kim et al., 2006; Wang et al., 2011; Kang and Kim, 2015). Similarly, the nationwide survey showed that prevalence rate of migraine in the general population was reported to be lower in Japan than other European countries (Rasmussen et al., 1991; Gobel et al., 1994; Sakai and Igarashi, 1997); Japan appears midway in the prevalence rate scale of migraines between European and other Asian countries. In terms of past histories, the presence of vascular risk factors with hypertension and/or diabetes mellitus had a lower tendency than other European and Asian populations (Peters et al., 2004; Singhal et al., 2004; Herve et al., 2009; Ciolli et al., 2014; Bianchi et al., 2015; Kang and Kim, 2015; Nannucci et al., 2018). The prevalence rate of dyslipidemia was in the range of previous reports (Peters et al., 2004; Herve et al., 2009; Adib-Samii et al., 2010; Ciolli et al., 2014; Kang and Kim, 2015; Nannucci et al., 2018; Koizumi et al., 2019). The prevalence rate of hypertension and diabetes mellitus in Japan (14.8 and 1.1%) seems to be much lower than the other countries (20–35.6% and 3–12.7%) (Peters et al., 2004; Singhal et al., 2004; Herve et al., 2009; Adib-Samii et al., 2010; Ciolli et al., 2014; Bianchi et al., 2015; Kang and Kim, 2015; Nannucci et al., 2018) similar to the previous report (Mizuta et al., 2017). The mean age of onset in Japanese CADASIL patients is within the range of other countries (42.7–52 years old) (Peters et al., 2004; Kim et al., 2006; Herve et al., 2009; Wang et al., 2011; Pescini et al., 2012; Ciolli et al., 2014; Kang and Kim, 2015).

**TABLE 3:** Comparison between Japanese CADASIL patients and other worldwide populations.

		Patient number and sex			Past History				Clinical presentation		MRI findings		Treatment
References	Countries	*n*	Onset (mean age)	Male sex (%)	Migraine (%)	HTN (%)	DL (%)	DM (%)	Stroke like episodes/Stroke (%)	Dementia (%)	Anterior temporal lobe lesion (%)	Microbleeds (%)	Anti-thrombotic medication (%)
Peters et al., 2004	Germany	80	45.7	46.3	34	25	23	3	78	44	NA	NA	75
Singhal et al., 2004	United Kingdom	127	NA	39.6	NA	20	9	4	NA	NA	NA	NA	NA
Herve et al., 2009	Germany and France	113	52	40.7	NA	19.5	41.9	3.6	NA	NA	NA	NA	NA
Adib-Samii et al., 2010	United Kingdom	200	33.6*	43	75.3	23.9	68.6	NA	NA	NA	NA	NA	NA
Pescini et al., 2012	Italy	61	50.7	49	49	NA	NA	NA	80	57	77	NA	NA
Ciolli et al., 2014	Italy	51	50.3	47.1	47.1	35.3	36	7.8	41.2	41.3	NA	NA	NA
Bianchi et al., 2015	Italy	229	NA	51	42	35.6	15.8	12.7	59	38	NA	NA	NA
Nannucci et al., 2018	Italy and United Kingdom	125	NA	45	67	30	58	6	NA	NA	88	34	70
Wang et al., 2011	China	33	42.7	51.5	5	NA	NA	NA	82	60	46	NA	NA
Kim et al., 2006	South Korea	40	47.7	55.6	4	NA	NA	NA	NA	NA	NA	NA	33.3
Kang and Kim, 2015	South Korea	49	52.7	43.5	8.7	21.7	60.9	6.3	NA	NA	NA	34.8	NA
Ueda et al., 2015	Japan	51	44.2–53.6	52.9	33	NA	NA	NA	69	31	70.6	NA	NA
Koizumi et al., 2019	Japan	126	NA	47.6	44.3	16.1	26.2	4.9	70.6	46.8	79.2	54.3	NA
Present study	Japan	88	49.5	62.5	13.6**	14.8	35.2	1.1	67	35.2	84.9	47.7	73.9

*HTN, hypertension; DL, dyslipidemia; DM, diabetes mellitus. ^∗^migraine age at onset. ^∗∗^migraine with and without aura was 28.4%.*

The prevalence rate of CADASIL was slightly lower than in other countries. Razvi et al. (2005) showed that the prevalence of CADASIL was 1.98 per 100,000 adults in Scotland, and Bianchi et al. (2015) described the prevalence at 4.1 per 100,000 adults in Italy. Uchino et al. (2002) reported that CADASIL was found in one of 2,030 stroke patients. It had been estimated that the incidence of new stroke was 220,000 per year in Japan (Takashima et al., 2017). Although, the number of patients with CADASIL expected to increase by approximately 108 per year from previous reports, we could identify only 88 patients in this study. There is a high possibility that the recognition of CADASIL varies depending on the facility, and there might be many undetected cases. Moreover, our study indicates that there is a regional difference in prevalence in Japanese CADASIL patients. Kansai, Kanto, and Kyusyu/Okinawa region have the three largest areas of the distribution of CADASIL patients. Note that we sent the questionnaire to all regions of Japan, and moreover, got replies from them all. Kanto region has the largest population, followed by Kansai, Chubu, and the Kyusyu/Okinawa region according to the 2015 data of Statistic Bureau, Ministry of Internal Affairs and Communications. Our study showed that Kansai region was the largest area in terms of distribution of CADASIL, indicating a predominance in the western part of Japan. In accordance with this observation, Ueda et al. (2015) reported a similar distribution of CADASIL patients in Japan and reported fewer numbers of CADASIL patients in the Hokkaido and Tohoku region. These results might depend on recognizing CADASIL itself in some hospitals in Japan.

The majority of *NOTCH3* gene mutations existed in exon 4 in Japan. This result is similar to the previous Japanese reports (Ueda et al., 2015; Mizuta et al., 2017; Koizumi et al., 2019). Previous studies showed a high frequency of *NOTCH3* gene mutation in exon 4 in Asian and European populations except Italy (Markus et al., 2002; Peters et al., 2004; Adib-Samii et al., 2010; Wang et al., 2011; Bianchi et al., 2015). Our study showed a lower prevalence of *NOTCH3* mutations in exon 3, especially the R75P, than previous reports in Japan (Ueda et al., 2015; Mizuta et al., 2017; Koizumi et al., 2019).

Although we conducted the first nationwide survey of CADASIL patients in Japan and constructed a registration database of CADASIL, we acknowledge that there are several limitations to our study. First, the number of the patients included in this study were small. Second, the prevalence rate of CADASIL might be lower than expected. However, this limitation may be related to the approach used by our survey study: we sent questionnaires to the departments of neurology of certificated hospitals. Thus, we might have missed patients treated by other departments or clinics. Moreover, there is a possibility that asymptomatic patients or those with only minor symptoms could not be identified. Therefore, the number of individuals with *NOTCH3* mutations might be underestimated. An alternative strategy involves asking genetic testing facilities for the number of *NOTCH3* mutation-positive individuals, which would ensure the inclusion of all the possible cases of *NOTCH3* mutation and thus, guarantee a larger study population. However, genetic testing for CADASIL is performed at several independent institutions, which might make it difficult to include all the positives in a single study. Therefore, we sent the questionnaire survey to hospitals for this study. This study sought the characteristics of CADASIL using a simple, two-step questionnaire, and therefore, detailed information may be lacking. However, through this study, we conducted a large survey involving more than one thousand certificated hospitals and created a novel registration database for future studies. We hope this study enables the increased recognition of CADASIL in Japan.

## Conclusion

This is the first nationwide survey of CADASIL in Japan. We have constructed the REDCap database for CADASIL patients in Japan. This database could be expanded and used for future clinical trials.

## Data Availability Statement

The raw data supporting the conclusions of this article will be made available by the authors, without undue reservation, to any qualified researcher.

## Ethics Statement

The studies involving human participants were reviewed and approved by the Ethics Committee of the Mie University Graduate School of Medicine (permit number 2918). Written consent was obtained from the patients who were registered in REDCap survey.

## Author Contributions

AS: draft of the manuscript, study concept and design, and acquisition of the data and analysis. KT, AT, TK, IM, MI, and TM: revision of the manuscript, interpretation of the data, and study supervision. TH, HW, MT, and SS: revision of the manuscript and interpretation of the data. HN, OO, AU, YA, TU, KKim, KKit, HH, HF, YM, and AW-H: acquisition of the data and interpretation of the data. HT: revision of the manuscript, study concept and design, and study supervision. All authors contributed to the article and approved the submitted version.

## Conflict of Interest

The authors declare that the research was conducted in the absence of any commercial or financial relationships that could be construed as a potential conflict of interest.
